# First record of *Viminella* sp. (Anthozoa: Alcyonacea: Ellisellidae) in the Persian Gulf

**DOI:** 10.3897/BDJ.7.e33089

**Published:** 2019-06-18

**Authors:** Shemshad Shahbazi, Nasrin Sakhaei, Hossein Zolgharnein, Catherine S McFadden

**Affiliations:** 1 Department of Marine Biology, Khorramshahr University of Marine Science and Technology, Khorramshahr, Iran Department of Marine Biology, Khorramshahr University of Marine Science and Technology Khorramshahr Iran; 2 1Department of Marine Biology, Khorramshahr University of Marine Science and Technology, Khorramshahr, Iran 1Department of Marine Biology, Khorramshahr University of Marine Science and Technology Khorramshahr Iran; 3 Department of Biology, Harvey Mudd College, Claremont, CA, United States of America Department of Biology, Harvey Mudd College Claremont, CA United States of America

**Keywords:** Octocorallia, gorgonian, Sclerite, Hengam Island, Iranian waters

## Abstract

This paper presents the first record of *Viminella* Gray 1870 from the Persian Gulf. This genus has a global distribution and its largest distribution is in the Indo-Pacific region. A single specimen was collected by a local fisherman from south Hengam Island (the north Persian Gulf) from 60-70 m depth. Colony morphology characteristics were examined by stereomicroscope and optical microscope. Descriptions of the colony and other taxonomic characteristics are provided. The colony is unbranched with a height of 12 cm and its basal diameter is approximately 1.63 mm. The sclerites of the coenenchyme comprise a variety of symmetrical double heads and capstan-like forms (0.05-0.11 mm). In the wall of calyces, slightly elongated double heads, capstans and spindles (about 0.08-0.19 mm) are present. Our finding extends the known geographical distribution of this genus in the Indo-Pacific region to the Persian Gulf.

## Introduction

Octocorals (Octocorallia: Alcyonacea: Alcyoniina) are globally distributed and are important elements of coral reef ecosystems, especially in the Indo-Pacific region. There are 35 genera of octocorals which are distributed over 15% of the region (Fabricius and De’ath 2000; Fabricius and Alderslade 2001; Chanmethakul et al. 2010). In the last decade, knowledge of the taxonomy of octocorals, especially from shallow tropical reefs in the Indo-Pacific region, has increased (Chanmethakul et al. 2010).

The Persian Gulf is one of the most important marine basins adjacent to the Indo-Pacific region and is distinguished by its unique biodiversity. As a marginal, shallow, semi-closed sea, it has immense economic and ecological value. The length of the Gulf is 990 km with a maximum recorded width of 240 km and the surface area is approximately 239,000 square km. Its mean depth is close to 36 m and, in some areas can be as deep as 100 m (Reynolds 1993). Thomson and Simpson (1909) were the first to study the taxonomy of octocorals in the Persian Gulf, reporting five species occurring in the region, *Solenocaulon tortuosum* (Gray, 1862), *Echinogorgia ramulosa* Gray, 1870, *Versluysia ramosa* (Thomson & Henderson, 1905), *Parisis fruticosa* Verrill, 1864 and a new species *Nicella reticulata* (a species currently in *Verrucella*). Recently Namin and Ofwegen (2009) identified 31 species from the Iranian coasts.

The genus *Viminella* (Octocorallia: Ellisellidae) was established by Gray (1870). It is a flagelliform gorgonian, characterised by unbranched colonies up to 2 m in height (Fabricius and Alderslade 2001; Williams and Chen 2011) and rarely with one or a few branches (Bayer and Grasshoff 1994; Fabricius and Alderslade 2001). Its colony colour is variable and can be red, orange, yellow, white, pink or bicoloured with red polyps or red with white polyps (Fabricius and Alderslade 2001; Williams and Chen 2011). This genus is widespread with a circumglobal distribution in the Indo-Pacific, Atlantic and Mediterranean Sea (Grasshoff 2000; Fabricius and Alderslade 2001; Kumar et al. 2014; Williams and Chen 2011). Fifteen species are known in this genus (Williams and Chen 2011). So far, *Viminella* has not been reported from the Persian Gulf.

## Materials and methods

**Figure 1.**
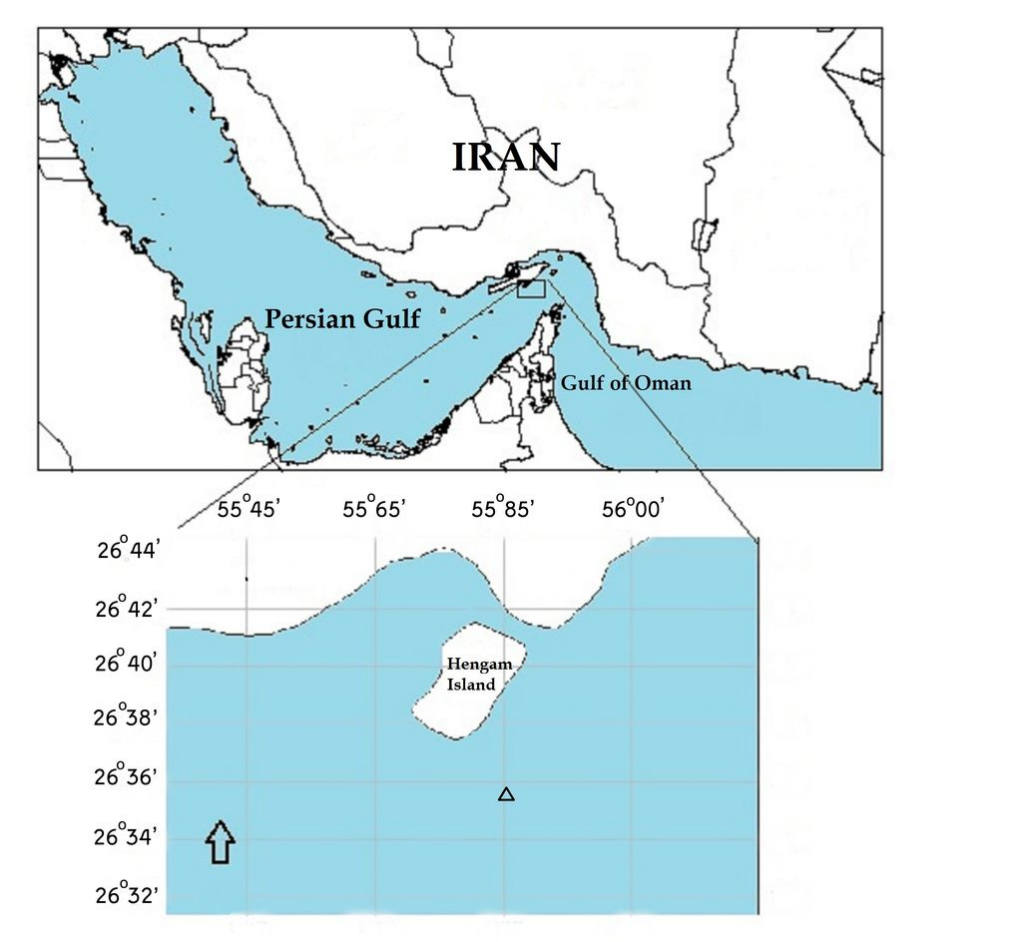
Sample collection site in the south coastal waters of the Hengam Island by local fishermen.

A colony of this specimen was collected by a local fisherman (with Gill net) from the north Persian Gulf area near Hengam Island (26°36'42.6''N 55°51'46.3''E) from 60-70 m depth (Fig. 1). The specimen was identified using morphological characteristics of the colony and sclerites. Sclerites were extracted using 5% sodium hypochlorite and examined under stereomicroscope and light microscope. The latest related references were used to identify this species (Bayer and Grasshoff 1994; Grasshoff 2000; Fabricius and Alderslade 2001; Kumar et al. 2014; Williams and Chen 2011). The colony, preserved in 75% ethanol, was deposited in the Khoramshahr University of Marine Science and Technology (KMSU).

## Results and Discussion

Order Alcyonacea Lamouroux, 1812

Suborder Calcaxonia Grasshoff, 1999

Family Ellisellidae Gray, 1859

Genus *Viminella* Gray, 1870

*Viminella* sp.

**Description**

**Figure 2.**
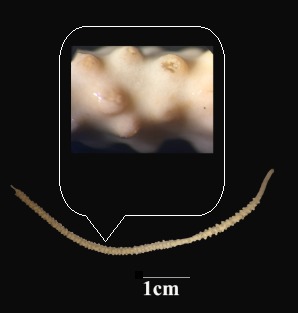
Wet-preserved specimen of *Viminella* sp. (KMSU).

**Figure 3.**
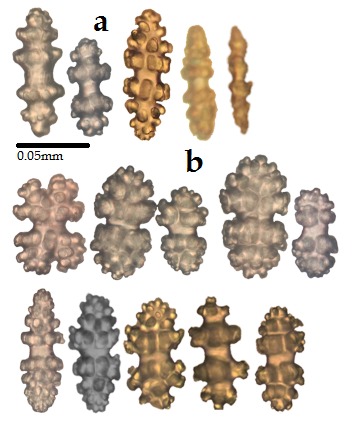
Sclerites of *Viminella* sp.: **a.** Polyp; **b.** Coenenchyme.

One colony of *Viminella* sp., found at a depth between 60 and 70 m, was examined. Colony unbranched with a height of 12 cm; basal diameter approximately 1.63 mm. Branch covered by monomorphic polyps. Polyps contract into conspicuous mound-like calyces (Fig. 2). Length of calyces about 0.45 mm. Sclerites symmetrical double heads and spindles (Fig. 3), light brown and highly tuberculated. Sclerites of coenenchyme a variety of symmetrical double heads and capstan-like forms (0.05-0.11 mm). In the wall of calyces, slightly elongated double heads, capstans and spindles (0.08-0.19 mm) present.

The genus *Viminella* occurs in shallow water as well as in deep water regions of the Indo-Pacific (Grasshoff and Bargibant 2001), but so far, it has not been reported from the Persian Gulf. In the Indo-Pacific region, *Viminella* is very similar to some species of *Junceella*. They have the same unbranched colony form but are easily identifiable by an investigation of the sclerites (Fabricius and Alderslade 2001). *Junceella* has non-symmetrical double-headed sclerites while *Viminella* has symmetrical double-headed sclerites.

## Hosting institution

Department of Marine Biology, Khorramshahr University of Marine Science and Technology, Khorramshahr , Iran

## References

[B4977582] Bayer F. M., Grasshoff M. (1994). The genus group taxa of the family Ellisellidae, with clarification of the genera established by JE Gray (Cnidaria; Octocorallia). Senckenbergiana Biologica.

[B4977592] Chanmethakul T., Chansang H., Watanasit S. (2010). Soft coral (Cnidaria: Alcyonacea) distribution patterns in Thai waters. Zoological Studies.

[B4978117] Fabricius Katharina, De’ath Glenn, Wolanski E J (2000). Biodiversity on the Great Barrier Reef: Large-scale patterns and turbidity-related local loss of soft coral taxa. Oceanographic Processes of Coral Reefs: Physical and Biological Links in the Great Barrier Reef.

[B4977602] Fabricius K., Alderslade P. (2001). Soft corals and sea fans: a comprehensive guide to the tropical shallow water genera of the central-west Pacific, the Indian Ocean and the Red Sea. Australian Institute of Marine Science.

[B4977630] Grasshoff M. (2000). The Gorgonians of the Sinai Coast and the Strait of Gubal, Red Sea (Coelanterata, Ostocorallia). Courier Forschungsinstitut Senckenberg.

[B4977621] Grasshoff M., Bargibant G. (2001). Coral Reef Gorgonians of New Caledonia. Collection Faune et Flore Tropicales 38.

[B4978054] Kumar Y., Raghunathan J. S., Raghuraman R, Sreeraj C. R., Venkataraman K. (2014). Gorgonians (Octocorallia) of Andaman and Nicobar Islands.

[B4977655] Namin K. S., Ofwegen L. (2009). Some shallow water octocorals (Coelenterata: Anthozoa) of the Persian Gulf. Zootaxa.

[B5000159] Reynolds R. M. (1993). Physical oceanography of the Gulf, Strait of Hormuz, and the Gulf of Oman—Results from the Mt Mitchell expedition. Marine Pollution Bulletin.

[B4977665] Thomson J. A., Simpson J. J. (1909). An Account of the Alcyonarians Collected by the Royal Indian Marine Survey Ship Investigator in the Indian Ocean; with a Report on the Species of *Dendronephthya* by W.D. Henderson. II. The Alcyonarians of the Littoral Area. The Indian Museum, Calcutta.

[B4977695] Williams GC, Chen J, Williams GC, Gosliner T (2011). Illustrated key to the shallow-water gorgonians and pennatulaceans of the Verde Island Passage, northern Philippines, including synopses of the taxa and a glossary of terms (Cnidaria: Anthozoa: Octocorallia).. The Coral Triangle: The 2011 Hearst Philippine Biodiversity Expedition.

